# Synthesis and Application of *N*-methylphthalimidylazo Disperse Dyes to Cellulose Diacetate for High Wash Fastness

**DOI:** 10.3390/ma13214924

**Published:** 2020-11-02

**Authors:** Sanghyun Yoon, Hyunjung Kim, Eunkyo Lee, Nahyun Oh, Sangcheon Kim, Kyung Hwa Hong, Joonseok Koh

**Affiliations:** 1 Department of Organic and Nano System Engineering, Konkuk University, 120 Neungdong-ro, Gwangjin-gu, Seoul 05029, Korea; sucre92@naver.com (S.Y.); bona111491@naver.com (H.K.); dldmsry1995@naver.com (E.L.); gina444@naver.com (N.O.); poi248@naver.com (S.K.); 2 Department of Fashion Design and Merchandising, Kongju National University, Chungnam 32588, Korea; hkh713@kongju.ac.kr; 3 Division of Chemical Engineering, Konkuk University, 120 Neungdong-ro, Gwangjin-gu, Seoul 05029, Korea

**Keywords:** phthalimide, disperse dyes, cellulose diacetate, build-up property, color fastness property, wash-off property

## Abstract

Cellulose diacetate fibers were prepared from cellulosic biomass with high α-cellulose contents such as purified cotton linters and wood pulps. Cellulose diacetate fibers are sensitive to alkaline solution, which causes hydrolysis of the acetate ester to hydroxyl groups, especially at high temperatures. Thus, the low alkali-resistance of cellulose acetate fibers makes it difficult to achieve high wash fastness by restricting the application of intense after-treatment, such as reduction clearing. A series of *N*-methylphthalimide-based high-washable azo disperse dyes were synthesized and their dyeing and fastness properties on cellulose diacetate fabrics were investigated. From the overall results obtained in this study, *N*-methylphthalimidylazo disperse dyes are expected to be a desirable alternative to high value-added dyes that can be used for high color fastness dyeing of cellulose diacetate with a minimal discharge of wastewater during washing process.

## 1. Introduction

**Figure 1:**
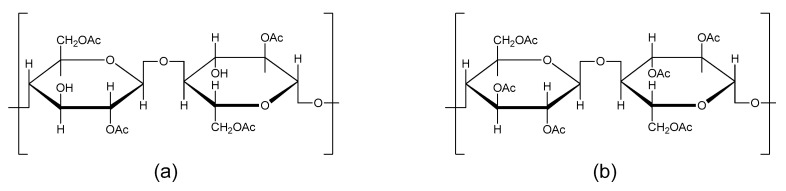
Chemical structures of acetate fibers: (**a**) Cellulose diacetate. (**b**) Cellulose triacetate (Ac = CH_3_CO).

Cellulose and its derivatives are attracting increasing attention as renewable, economical, and abundant resources to reduce dependency on fossil resources [1,2,3]. Cellulose acetate has been recognized as by far the most important derivative of cellulose because of its versatile industrial and commercial applications [4,5]. Acetate fibers are synthetic cellulosic fibers manufactured by treating cellulosic biomass, such as pure wood pulp or cotton linters, with a mixture of acetic anhydride and acetic acid at a low temperature in the presence of a catalyst such as sulfuric acid, perchloric acid, zinc chloride, or similar salts [6]. The cellulose hydroxyl groups are esterified by the acetic anhydride to form cellulose acetates—the fiber forming components. Cellulose acetate is majorly classified into two types: cellulose triacetate and cellulose diacetate, with acetylation degrees of 3.00 and 2.48, respectively (Figure 1) [7,8].

**Figure 2:**
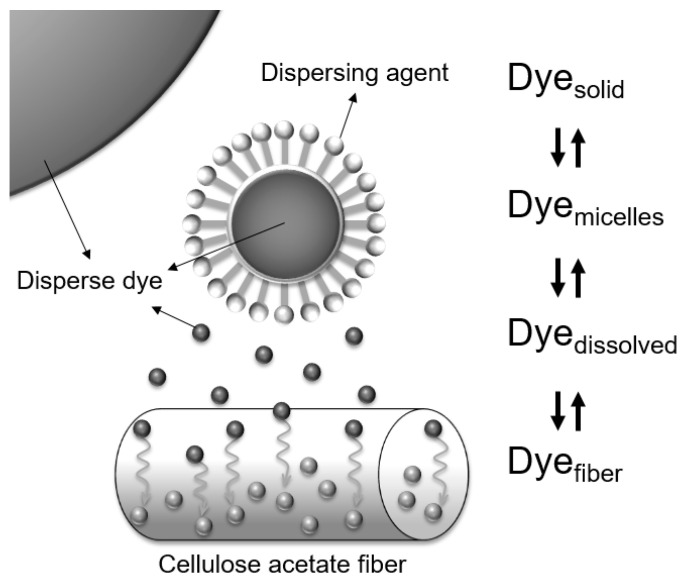
Disperse dyeing mechanism of cellulose acetate fiber.

Cellulose diacetate fibers are widely used in the apparel industry because of their superior properties, such as silk-like softness, luster, comfort, and good draping quality. However, dyes with satisfactory properties—non-toxicity, environmentally friendliness, and high color fastness, have been a major impediment in their use in the textile industry. Due to their hydrophobic nature, efficient dyeing requires the use of dyes with limited water solubility; disperse dyes are sparingly soluble in water and are almost exclusively used for cellulose diacetate fibers. The insolubility of disperse dyes enables them to transfer from water to the surface of hydrophobic fibers, and the application of heat to the dye liquor accelerates the disperse dye’s diffusion into the interior of the fiber above its glass transition temperature (Figure 2) [8].

**Scheme 1:**
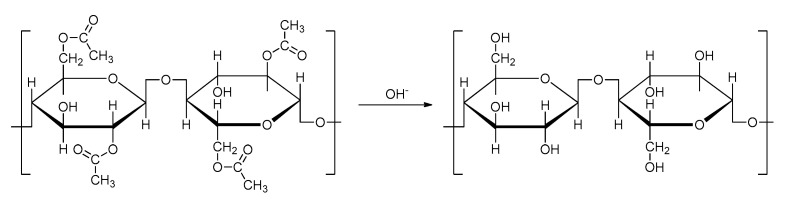
Alkali-hydrolysis of cellulose diacetate fibers.

While the development of disperse dyes was a major breakthrough, their use for cellulose diacetate fibers is faced with numerous challenges and they are consequently limited as compared to other fabrics. To achieve optimal color fastness during disperse dyeing of polyester fiber, excess dye on the fiber surface is cleared by reductive treatment in a strong reduction bath made up of a toxic reducing agent (i.e., sodium hydrosulfite) and a strong alkali (i.e., sodium hydroxide) [9]. However, this is not achievable with cellulose diacetate. During reduction-clearing, the acetyl groups are hydrolyzed, leading to the deterioration of their characteristic properties (Scheme 1). Alternatively, the dyed acetate fabrics are aftertreated with soaping agents at a relatively low temperature. Although this method retains their unique characteristics, the resultant color fastness properties are not satisfactory [8]. In this case, fastness can be improved by washing several times using large amounts of wash water, which consequently increases the wastewater load in the dyehouse effluent.

**Scheme 2:**
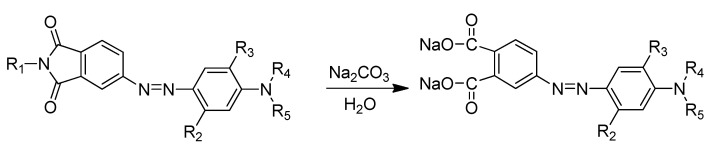
Alkali-hydrolysis of phthalimidylazo disperse dyes under milk alkaline condition (Na_2_CO_3_ 1.0 g/L, 80 °C for 20 min; R^1^ = alkyl, phenyl; R^2^, R^3^ = H, CH_3_, OCH_3_, NHCOCH_3_; R^4^, R^5^ = substituted alkyls).

In our previous study, we demonstrated phthalimidylazo-based alkali-clearable disperse dyes as a solution to environmental problems in the polyester dyeing industry, which subsequently eliminated the need for a toxic reducing agent and a strong alkali, thereby reducing the cost of wastewater treatment [10]. Azo disperse dyes incorporating a phthalimide group hydrolyze to dyes containing a water-solubilizing diester group under relatively mild alkaline to neutral conditions without causing cleavage of the azo bond and are easily washed-off (Scheme 2). The hydrolysis mechanism of phthalimidylazo disperse dyes was verified through analysis of the dye hydrolysis under mild alkali conditions by FT-IR (Fourier-transform infrared), mass, and UV- Vis (Ultraviolet-visible) spectrophotometry. HPLC (High performance liquid chromatography) analysis showed that mild alkaline treatment (Na_2_CO_3_ 1.0 g/L) of phthalimidylazo disperse dyes at 80 °C for 20 min resulted in more than 95% hydrolysis of parent dyes and the overall color fastness of dyed polyester was in the range of 4–5 to 5 [10,11]. However, the phthalimidylazo disperse dyes showed excellent stability under the conventional high temperature disperse dyeing condition. i.e., less than 3% hydrolysis during dyeing at 130 °C for 60 min (pH 4.5). Therefore, the use of highly washable phthalimidylazo disperse dyes is expected to ensure excellent stability during dyeing and improved color fastness properties of cellulose diacetate fibers with a mild aftertreatment, such as soaping treatment, without compromising their unique characteristics.

In the present study, *N*-methylphthalimide-based highly washable azo disperse dyes were synthesized and their dyeing and fastness properties on cellulose acetate fabrics were compared with those of typical 4-aminoazobenzene disperse dyes. Specifically, in order to increase the affinity of phthalimidylazo disperse dyes for cellulose diacetate fibers having a relatively less hydrophobic nature than polyester fibers, hydrophilic substituents were introduced into the coupling component of the dyes.

## 2. Materials and Methods

### 2.1. Materials and Reagents

The chemicals used in the synthesis of disperse dyes were *N* -methyl-4-aminophthalimide, *N*, *N* -diethylaniline, 2-(*N*-ethylanilino)ethanol, *N*-phenyldiethanolamine, *N*, *N*-diethyl-m-toluidine, 2-(*N* -ethyl-m-toluidino)ethanol, m-tolyldiethanolamine, *N*, *N*-dimethylformamide, dimethyl sulfoxide-d6 (DMSO-*d6*), sodium nitrite, sodium acetate, and methanol. Cellulose diacetate fabrics (70 ± 5 g/m^2^; plain weave, warp, and weft each 75 denier) were used for disperse dyeing. All chemicals used for the synthesis and dyeing were of laboratory reagent grade. Dispersing agent (Diwatex, Borregaard Lignotech, anionic, Sarpsborg, Norway) and wetting agents (Sandozin NIE, Clariant, nonionic, Muttenz, Switzerland) were used for milling. Lyocol RDN liquid (Clariant, anionic, Muttenz, Switzerland) was used as a dispersing agent in dyeing experiments. Inkanol AZ-100 (Poong Yeong Chem, nonion, Gyeonggi-do, Korea) was used as a soaping agent in aftertreatment.

### 2.2. Instrumentation

The IR spectra were obtained with a FT-IR Spectrum 2000 (16 scans) spectrophotometer (Perkin Elmer, Waltham, MA, USA). ^1^H-NMR (Nuclear magnetic resonance) spectra were obtained using an Avance 500 (Bruker, Karlsruhe, Germany) at 500 MHz for solutions in DMSO-*d_6_*. Elemental analyses were carried out on a CHNS-932 (LECO Corporation, St. Joseph, MI, USA) for C, H, N, and S. The melting point was measured using an M-560 melting point instrument (Buchi, Flawil, Switzerland). The absorption spectra were measured in 1 cm quartz cells on an HP8453 UV-visible spectrophotometer (Agilent, Santa Clara, CA, USA). The reflectance and color properties (CIE L*, a*, b*, C*, and h*_ab_*) of the dyed samples were measured using an X-Rite 8000 Series (X-Rite, Inc., Grand Rapids, MI, USA) in specific color measurement conditions (standard light D65, 10° standard observer, and specular component included).

### 2.3. Dye Synthesis and Preparation of Dye Dispersions

**Scheme 3:**
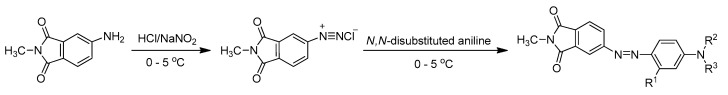
Synthesis of azo disperse dye containing an *N*-methylphthalimide group.

Syntheses of *N*-methylphthalimidylazo dyes (P-dyes, P1-P6) (Scheme 3) and 4-nitroaminoazobenzene dyes (N-dyes, N1-N6) were carried out as described in the previous studies (Table 1) [12,13]. In total, 0.02 mol of diazo component was diazotized in 6.9 mL of 35% HCl and 72 mL of water by adding 0.02 mol of NaNO_2_ at a temperature of 0–5 °C. After 5–6 h, the solution of diazonium salt was added to a solution containing 0.02 mol of coupling component, 40 mL of water, and 3.4 mL of 35% HCl. After 5–6 h, the precipitated dye was filtered, washed with water, and dried. The dyes were purified by crystallization from methanol. The structural analysis and characterization data of the synthesized dyes are given below.

P1. (5-((4-(diethylamino)phenyl)diazenyl)-2-methylisoindoline-1,3-dione). Reddish orange solid. Yield 75.2%. M.p. 187–188 °C. IR (KBr) ν_max_: 2978 (w, CH), 1715 (s, C = O), 1600, 1518 (s, ArC = C), 1557 (w, N = N) cm^−1^. ^1^H NMR (500 MHz, DMSO-d_6_) δ: 1.17 (6H, t, *J* = 7.2, N(CH_2_CH_3_)_2_), 3.06 (3H, s, pthalimide-CH_3_), 3.50 (4H, q, *J* = 7.0, N(CH_2_CH_3_)_2_), 6.84 (2H, d, *J* = 9.5, ArH), 7.84 (2H, d, *J* = 9.5, ArH), 7.93 (1H, d, *J* = 9.5, phthalimide-H), 8.00 (1H, s, phthalimide-H), 8.12 (1H, d, *J* = 8.0, phthalimide-H) ppm. Anal. calcd for C_19_H_20_N_4_O_2_ (%): C 67.84; H 5.99; N 16.66, found: C 67.69; H 5.95; N 16.61.
P2. (5-((4-(ethyl(2-hydroxyethyl)amino)phenyl)diazenyl)-2-methylisoindoline-1,3-dione). Reddish orange solid. Yield 62.3%. M.p. 196–197 °C. IR (KBr) ν_max_: 3239 (s, OH), 2978 (w, CH), 1714 (s, C = O), 1600, 1518 (s, ArC = C), 1555 (w, N = N), 1080 (m, C-O) cm^−1^. ^1^H NMR (500 MHz, DMSO-d_6_) δ: 1.16 (3H, t, *J* = 7.0, NCH_2_CH_3_), 3.05 (3H, s, phthalimide-CH_3_), 3.50–3.56 (4H, m, NCH_2_), 3.631 (2H, t, *J* = 5.9, NCH_2_CH_2_OH), 4.84 (1H, s, NCH_2_CH_2_OH), 6.87 (2H, d, *J* = 9.0, ArH), 7.82 (2H, d, *J* = 9.0, ArH), 7.93 (1H, d, *J* = 8.0, phthalimide-H), 8.00 (1H, s, phthalimide-H), 8.11 (1H, d, *J* = 8.0, phthalimide-H) ppm. Anal. calcd for C_19_H_20_N_4_O_3_ (%): C 64.76; H 5.72; N 15.90, found: C 64.58; H 5.69; N 15.86.
P3. (5-((4-(bis(2-hydroxyethyl)amino)phenyl)diazenyl)-2-methylisoindoline-1,3-dione). Reddish orange solid. Yield 59.4%. M.p. 207–208 °C. IR (KBr) ν_max_: 3239 (s, OH), 2978 (w, CH), 1709 (s, C = O), 1600, 1518 (s, ArC = C), 1560 (w, N = N), 1042 (m, C-O) cm^−1^. ^1^H NMR (500 MHz, DMSO-d_6_) δ: 3.06 (3H, s, phthalimide-CH_3_), 3.59–3.63 (8H, m, NCH_2_CH_2_), 4.86 (2H, s, N(CH_2_CH_2_OH)_2_), 6.90 (2H, d, *J* = 9.3, ArH), 7.83 (2H, d, *J* = 9.1, ArH), 7.93 (1H, d, *J* = 8.0, phthalimide-H), 8.00 (1H, s, phthalimide-H), 8.11 (1H, d, *J* = 8.0, phthalimide-H). Anal. calcd for C_19_H_20_N_4_O_4_ (%): C 61.95; H 5.47; N 15.21, found: C 61.78; H 5.42; N 15.17.
P4. (5-((4-(diethylamino)-2-methylphenyl)diazenyl)-2-methylisoindoline-1,3-dione). Red powder. Yield 81.0%. M.p. 204–205 °C. IR (KBr) ν_max_: 2972 (w, CH), 1714 (s, C = O), 1600, 1511 (s, ArC = C), 1559 (w, N = N) cm^−1^. ^1^H NMR (500 MHz, DMSO-d_6_) δ: 1.18 (6H, t, *J* = 7.0, N(CH_2_CH_3_)_2_), 2.65 (3H, s, ArCH_3_), 3.06 (3H, s, phthalimide-CH_3_), 3.49 (4H, q, *J* 7.2, N(CH_2_CH_3_)_2_), 6.68 (2H, d, *J* = 7.3, ArH), 7.74 (2H, d, *J* = 9.9, ArH), 7.97 (1H, d, *J* = 7.9, phthalimide-H), 8.01 (1H, s, phthalimide-H), 8.10 (1H, d, *J* = 8.0, phthalimide-H). Anal. calcd for C_20_H_22_N_4_O_2_ (%): C 68.55; H 6.33; N 15.99, found: C 68.40; H 6.30; N 15.92.
P5. (5-((4-(ethyl(2-hydroxyethyl)amino)-2-methylphenyl)diazenyl)-2-methylisoindoline-1,3-dione). Red powder. Yield 67.2%. M.p. 210–211 °C. IR (KBr) ν_max_: 3200 (s, OH), 2978 (w, CH), 1714 (s, C = O), 1600, 1505 (s, ArC = C), 1557 (w, N = N), 1032 (me, C-O) cm^−1^. ^1^H NMR (500 MHz, DMSO-d_6_) δ: 1.16 (3H, t, *J* = 7.0, NCH_2_CH_3_), 2.63 (3H, s, ArCH_3_) 3.05 (3H, s, phthalimide-CH_3_), 3.49-3.54 (4H, m, NCH_2_), 3.61 (2H, t, *J* = 6.0, NCH_2_CH_2_-OH), 4.83 (1H, s, NCH_2_CH_2_OH), 6.69 (2H, m, ArH), 7.70 (1H, d, *J* = 9.9, ArH), 7.94 (1H, d, *J* = 7.8, phthalimide-H), 7.97 (1H, s, phthalimide-H), 8.07 (1H, d, *J* = 7.9, phthalimide-H). Anal. calcd for C_20_H_22_N_4_O_3_ (%): C 65.56; H 6.05; N 15.29, found: C 65.38; H 6.01; N 15.22.
P6. (5-((4-(bis(2-hydroxyethyl)amino)-2-methylphenyl)diazenyl)-2-methylisoindoline-1,3-dion). Reddish orange solid. Yield 62.3%. M.p. 221–222 °C. IR (KBr) ν_max_: 3226 (s, OH), 2988 (w, CH), 1714 (s, C = O), 1600, 1508 (s, ArC = C), 1566 (w, N = N), 1073 (m, C-O) cm^−1^. ^1^H NMR (500 MHz, DMSO-d_6_) δ: 2.63 (3H, s, ArCH_3_), 3.04 (3H, s, phthalimide-CH_3_), 3.56–3.61 (8H, m, N(CH_2_CH_2_OH)_2_), 4.85 (2H, s, N(CH_2_CH_2_OH)_2_), 6.70 (2H, d, *J* = 9.5, ArH), 7.68 (2H, d, *J* = 10.0, ArH), 7.94 (1H, d, *J* = 9.9, phthalimide-H), 7.96 (1H, s, phthalimide-H), 8.07 (1H, d, *J* = 7.9, phthalimide-H). Anal. calcd for C_20_H_22_N_4_O_4_ (%): C 62.82; H 5.80; N 14.65, found: C 62.65; H 5.79; N 14.60.
N1. (*N*,*N*-diethyl-4-((4-nitrophenyl)diazenyl)aniline). Brownish red solid. Yield 89.3%. M.p. 150–151 °C. IR (KBr) ν_max_: 2950 (w, CH), 1600, 1508 (s, ArC = C), 1556 (w, N = N), 1512, 1353 (s, NO_2_) cm^−1^. ^1^H NMR (500 MHz, DMSO-d_6_) δ: 1.17 (6H, t, *J* = 7.0, N(CH_2_CH_3_)_2_), 3.50 (4H, q, *J* = 7.2, N(CH_2_CH_3_)_2_), 6.85 (2H, d, *J* = 9.0, ArH), 7.84 (2H, d, *J* = 9.5, ArH), 7.92 (2H, d, *J* = 9.0, ArH), 8.35 (2H, d, *J* = 9.0, ArH). Anal. calcd for C_16_H_18_N_4_O_2_ (%): C 64.41; H 6.08; N 18.78, found: C 64.28; H 6.05; N 18.72.
N2. (2-(ethyl(4-((4-nitrophenyl)diazenyl)phenyl)amino)ethanol): dark brownish red solid; yield 66.2%. M.p. 159–160 °C. IR (KBr) ν_max_: 3429 (s, OH), 2937 (w, CH), 1600, 1508 (s, ArC = C), 1557 (w, N = N), 1512, 1353 (s, NO_2_), 1000 (m, C-O) cm^−1^. ^1^H NMR (500 MHz, DMSO-d_6_) δ: 1.17 (3H, t, *J* = 7.0, NCH_2_CH_3_), 3.51-3.55 (4H, m, NCH_2_), 3.63 (2H, t, *J* = 6.0, NCH_2_CH_2_OH), 4.86 (1H, s, NCH_2_CH_2_OH), 6.87 (2H, d, *J* = 9.0, ArH), 7.82 (2H, d, *J* = 9.0, ArH), 7.95 (2H, d, *J* = 8.0, ArH), 8.10 (2H, d, *J* = 8.0, ArH). Anal. calcd for C_16_H_18_N_4_O_3_ (%): C 61.13; H 5.77; N 17.82, found: C 60.94; H 5.72; N 17.76.
N3. (2,2’-((4-((4-nitrophenyl)diazenyl)phenyl)azanediyl)diethanol. Brownish red solid. Yield 60.8%. M.p. 170–171 °C. IR (KBr) ν_max_: 3390 (s, OH), 2975 (w, CH), 1600, 1508 (s, ArC = C), 1559 (w, N = N), 1512, 1333 (s, NO_2_), 1048 (m, C-O) cm^−1^. ^1^H NMR (500 MHz, DMSO-d_6_) δ: 3.62–3.67 (8H, m, N(CH_2_CH_2_OH) _2_), 4.89 (1H, s, N(CH_2_CH_2_OH)_2_), 6.91 (2H, d, *J* = 9.0, ArH), 7.82 (2H, d, *J* = 9.0, ArH), 7.91 (2H, d, *J* = 9.0, ArH), 8.34 (2H, d, *J* = 9.0, ArH). Anal. calcd for C_16_H_18_N_4_O_4_ (%): C 58.17; H 5.49; N 16.96, found: C 58.00; H 5.42; N 16.90.
N4. (*N*,*N*-diethyl-3-methyl-4-((4-nitrophenyl)diazenyl)aniline). Violet solid. Yield 64.4%. M.p. 164–165 °C. IR (KBr) ν_max_: 2928 (w, CH), 1600, 1508 (s, ArC = C), 1559 (w, N = N), 1512, 1359 (s, NO_2_) cm^−1^. ^1^H NMR (500 MHz, DMSO-d_6_) δ: 1.16 (6H, t, *J* = 7.0, N(CH_2_CH_3_)_2_), 2.65 (3H, s, ArCH_3_), 3.49 (4H, q, *J* = 7.2, N(CH_2_CH_3_)_2_), 6.85 (2H, d, *J* = 9.0, ArH), 7.74 (1H, d, *J* = 10.0, ArH), 7.97 (2H, d, *J* = 9.9, ArH), 8.10 (2H, d, *J* = 8.0, ArH). Anal. calcd for C_17_H_20_N_4_O_2_ (%): C 65.37; H 6.45; N 17.94, found: C 65.11; H 6.41; N 17.89.
N5. (2-(ethyl(3-methyl-4-((4-nitrophenyl)diazenyl)phenyl)amino)ethanol). Dark brownish red solid. Yield 58.9%. M.p. 173–174 °C. IR (KBr) ν_max_: 3271 (s, OH), 2975 (w, CH), 1603, 1508 (s, ArC = C), 1559 (w, N = N), 1512, 1355 (s, NO_2_), 1044 (m, C-O) cm^−1^. ^1^H NMR (500 MHz, DMSO-d_6_) δ: 1.16 (3H, t, *J* = 7.0, NCH_2_CH_3_), 2.63 (3H, s, ArCH_3_) 3.50–3.54 (4H, m, NCH_2_), 3.62 (2H, t, *J* = 6.0, NCH_2_CH_2_OH), 4.86 (1H, s, NCH_2_CH_2_OH), 6.69 (2H, d, *J* = 4.5, ArH), 7.70 (1H, d, *J* = 10.0, ArH), 7.94 (2H, d, *J* = 8.0, ArH), 8.08 (2H, d, *J* = 8.0, ArH). Anal. calcd for C_17_H_20_N_4_O_3_ (%): C 62.18; H 6.14; N 17.06, found: C 62.01; H 6.11; N 17.01.
N6. (2,2’-((3-methyl-4-((4-nitrophenyl)diazenyl)phenyl)azanediyl)diethanol). Dark brownish red solid. Yield 59.1%. M.p. 184–185 °C. IR (KBr) ν_max_: 3276 (s, OH), 2930 (w, CH), 1603, 1508 (s, ArC = C), 1558 (w, N = N), 1512, 1340 (s, NO_2_), 1067 (m, C-O) cm^−1^. ^1^H NMR (500 MHz, DMSO-d_6_) δ: 2.63 (3H, s, ArCH_3_), 3.59–3.63 (8H, m, N(CH_2_CH_2_OH)_2_), 4.85 (2H, s, N(CH_2_CH_2_OH)_2_), 6.70 (2H, d, *J* = 9.5, ArH), 7.68 (1H, d, *J* = 10.0, ArH), 7.93 (2H, d, *J* = 8.0, ArH), 8.072 (2H, d, *J* = 8.0, ArH). Anal. calcd for C_17_H_20_N_4_O_4_ (%): C 59.29; H 5.85; N 16.27, found: C 59.02; H 5.82; N 16.21.

Dye dispersion (1.0 *w*/*v* %) was prepared in water. Dispersing agent (Diwatex, 40% on weight of dye), wetting agent (Sandozin NIE Liquid, 1 drop), and 1.0 g of dye were milled with glass beads for 24 h in 100 mL water buffered at pH 4.0–4.5.

### 2.4. Dyeing

Cellulose diacetate fabrics were dyed in a laboratory dyeing machine (DL-6000; Daelim Starlet Co. Ltd., Gyeonggi-do, Korea) at a liquor ratio of 20:1. A 40 mL dyebath was prepared using a dispersing agent, formulated dye dispersion, and distilled water. The cellulose diacetate fabric (2.0 g) was dyed in the dyebath for 60 min at 85 °C and washed with soaping agent (1.0 g/L) for 15 min at 45 °C.

The build-up properties of the dyes on cellulose acetate were investigated by measuring the color strength of dyed fabrics at various dye concentrations. K/S values were calculated from the reflectance values according to Kubelka–Munk theory and the color strength (*f_k_*) was determined from the sum of the weighed K/S values in the visible region, given by [14]:

(1) $$f_{k}\;=\;\sum_{\lambda =400}^{700}{\left(K/S\right)}_{\lambda }({\bar{x}}_{10,\lambda }+{\bar{y}}_{10,\lambda }+{\bar{z}}_{10,\lambda })$$

where $\;{\bar{x}}_{10,\lambda }$, ${\bar{y}}_{10,\lambda }$, and ${\bar{z}}_{10,\lambda }$ are the color matching functions for the 10° standard observer at each wavelength (ISO 7724/1-1984).

### 2.5. Color Fastness Test

To evaluate the color fastness properties of the synthesized dyes on cellulose diacetate fabrics, the dyed fabric was subjected to three different types of aftertreatment i.e., soaping, a hot-rinse and no-treatment, and heat-set at 150 °C for 45 s in a laboratory tenter (Tex-dryer, Daelim Starlet Co. Ltd., Gyeonggi-do, Korea). 1/1 standard depth was used for the color fastness test. Wash fastness was determined using international standards (ISO 105-C06 D3S) [15]. Changes in shade and staining of adjacent multifiber (Multifiber DW, adjacent fabric, BS EN ISO 105-F10, SDC L Enterprises Limited, Holmfirth, UK) [15] were assessed using grey scales. A numerical grading was given on a scale of 1 to 5, where a higher number indicated better fastness.

## 3. Results

### 3.1. Spectral Properties of the Synthesized Dyes

**Figure 3:**
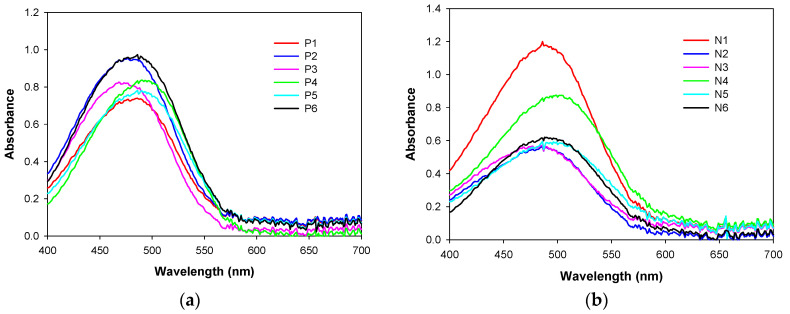
UV/Visible spectra of the synthesized dyes in ethanol: (**a**) P-dyes. (**b**) N-dyes.

As shown in Table 2, N-dye series exhibited higher extinction coefficients and half-band width, and therefore gave greater tinctorial strength as compared to P-dye series. The UV–Visible spectra of the synthesized dyes in ethanol solution are shown in Figure 3. Regarding the spectral properties of the synthesized dyes, the color ranges of P-dye series and N-dye series were almost identical, both of which fall into the color gamut of orange to red (477–499 nm). However, the extinction coefficient (ε_max_) and half band-width (Δλ_1/2_) were at different levels. Among the P-dye series, P2, P3, and P6 showed very similar extinction coefficients to their analogs in N-dye series. Therefore, we can expect P2, P3, and P6 will exhibit a relatively reasonable level of build-up properties. The value of half band-width is a useful criterion for the evaluation of the hue brightness of dyes [16]. Dyes with low half band-width have bright hues, while those with high half bandwidth have dull hues. Comparatively, P-dye series has lower half band-width than N-dye series; thus, they are expected to have higher chromaticity on the dyed cellulose diacetate.

### 3.2. Dyeing Properties on Cellulose Diacetate

#### 3.2.1. Buildup Properties on Cellulose Diacetate

**Figure 4:**
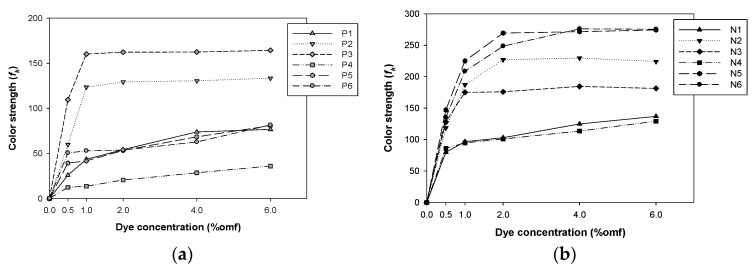
Build-up properties of the synthesized dyes on cellulose diacetate: (**a**) P-dyes. (**b**) N-dyes.

Figure 4 illustrates the relative color strength (*f_k_*) of dyed cellulose diacetate fabrics depending on the amount of dye applied (mass of fabric, %omf). P-dyes exhibited slower buildup and reduced color yield at saturation points compared to N-dyes. For example, the saturation points for P2 and P3 were between 1.0 and 2.0 %omf, while those of N2 and N3 were between 2.0 and 4.0 %omf. Thus, in dyeing cellulose diacetate fiber, the substantivity of *N*-methylphthalimidylazo dyes was shown to be lower than that of 4-aminoazobenzene dyes containing a *p*-nitro group. The relatively poor build-up properties of P-dye series can be explained by the relatively lower tinctorial strength and affinity to acetate due to the lower polarity. This explains why many commercial azo disperse dyes for PET (poly (ethylene terephthalate)) and acetate fibers contain the *p*-nitro group in the diazo component. Among the P-dye series, P3 showed the better build-up properties on cellulose diacetate compared to the other P-dyes probably because of its higher tinctorial strength and relatively lower hydrophobic character due to the inclusion of two hydrophobic hydroxy groups and no methyl group in its coupling component. In addition, the hydrogen bonding and polar interactions between hydroxyl groups of cellulose diacetate and the di(hydroxyethyl)amine moiety of the dye’s structure could contribute to the better dyeability of P3.

**Figure 5:**
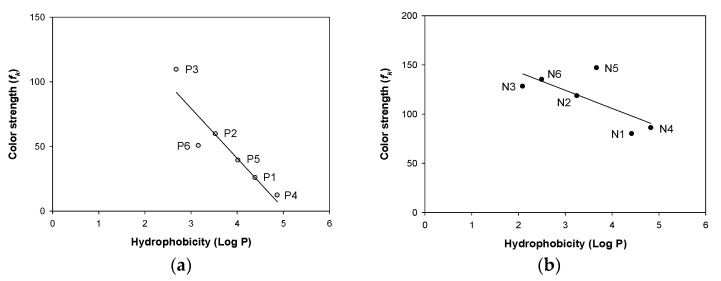
Correlation between hydrophobicity (log P) and color strength (*f_k_*) of the synthesized dyes on cellulose diacetate: (**a**) P-dyes. (**b**) N-dyes.

From the scatter plots showing the relationship between dyeability and hydrophobicity of dyes (Figure 5), it is clear that dyes with lower hydrophobicity exhibit better build-up properties on cellulose diacetate fibers. This is due to the relatively lower hydrophobic nature of cellulose acetate fibers compared to that of PET. P1, P4, N1, and N4 with the higher log P values (P1 = 4.39, P4 = 4.87, N1 = 4.42, N4 = 4.83) due to the hydrophobic *N*, *N*-diethyl group showing the lower build-up property on cellulose diacetate fibers. Cellulose acetate is not too hydrophobic, nor is it hydrophilic. This results in moisture-retention properties that relate to the excellent comfort factor [17].

#### 3.2.2. Color Properties on Cellulose Diacetate

**Figure 6:**
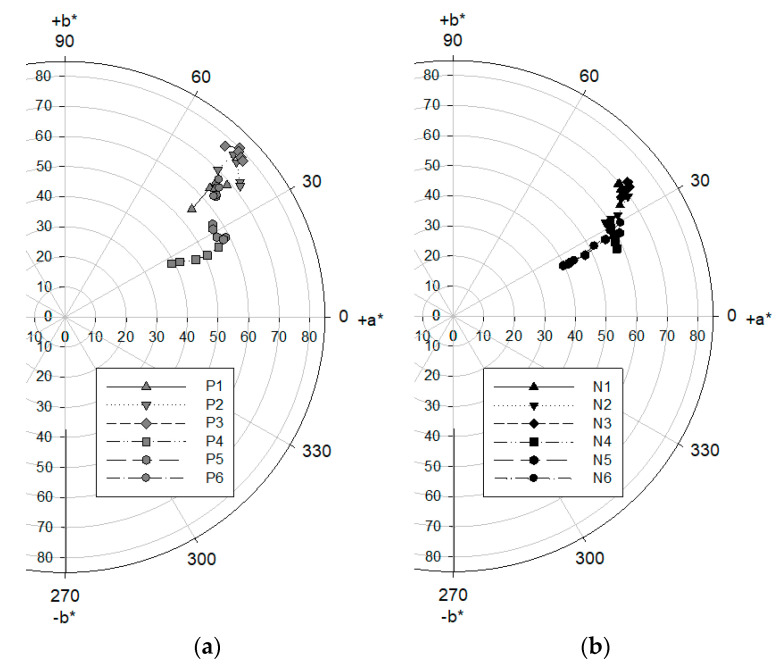
CIELAB color coordinates (a* and b*) of the cellulose diacetate fibers dyed with the synthesized dyes: (**a**) P-dyes. (**b**) N-dyes.

The colorimetric properties of dyes, expressed in terms of the CIE (Commission International d’Eclairage) 1976 L*a*b* color space parameters, as shown in Table 3, expresses the dyeing behavior of dyes. The colors of synthesized N-dyes and P-dyes on cellulose diacetate fall into the range of orange to red. P-dyes (P1–P6) show relatively higher chroma (C*) than N-dyes (N1–N6) (Figure 6). As expected, P-dyes were slightly hypsochromic on cellulose diacetate compared with N-dyes. This can be explained by the relatively lower electron-withdrawing power of the *N*-methylphthalimidyl group compared to 4-nitroanilne. These trends in colorimetric properties are consistent with their spectral properties in solvent, as shown in λ_max_ in Table 2 and h_ab_ in Table 3. Additionally, most P-dyes showed higher chromaticity values on the cellulose diacetate fibers compared to N-dyes. This property can be explained by their narrower half-band width values shown in Table 2. Color photographs of the dyed cellulose diacetate fabrics are shown in Table 4.

### 3.3. Color Fastness to Washing

**Figure 7:**
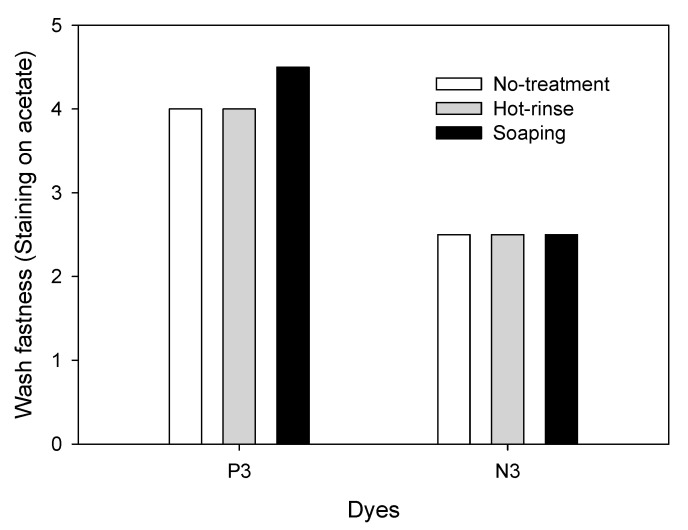
Wash fastness of cellulose diacetate fabrics dyed with dyes P3 and N3 at 1/1 standard depth dyeing.

Table 5 shows the wash fastness test result at 1/1 standard depth dyeing with N-dyes and P-dyes. For this test, P3 was chosen because the other P-dyes cannot reach the 1/1 standard depth even at the heaviest dyeing concentration due to their poor build-up properties. In order to compare their fastness properties, the analog of nitroaniline-based dyes N3 was chosen. The acetate and nylon components of adjacent multifiber fabrics are prone to staining during the wash fastness test of disperse-dyed cellulose diacetate fibers. In the case of the dyed cellulose diacetate fabrics being aftertreated with soaping, the staining on wool, acryl, PET, and cotton components of adjacent multifiber fabrics was in the range of 4–5 to 5 for P3 and 4 to 4–5 for N3, respectively. Not only the aftertreated sample with soaping but also non-treated and hot rinsed samples after dyeing exhibited similarly excellent levels of wash fastness, presumably because the phthalimide group of the synthesized dyes was hydrolyzed to solubilizing diester groups during soaping aftertreatment, which involves weak alkaline with a pH of around 9–10, as shown in previous studies [10,11]. The dye residues were, therefore, more readily removed during the wash-off and exhibited low staining on adjacent multifiber fabric in the wash fastness test. However, in the case of N3, even the aftertreated sample with soaping did not show an improved wash fastness level compared to non-treated and hot rinsed samples. An analogous comparison between P3 and N3 revealed a gap in wash-off property, exemplified by the staining of nylon (Figure 7). P3 exhibited higher wash fastness than N3 by as much as about 2.0 grading units, irrespective of the aftertreatment methods. The overall results show that *N*-methylphthalimidylazo dyes have excellent wash fastness on cellulose diacetate fibers compared to the conventional 4-nitro-4’-nitroazobenzene disperse dyes.

## 4. Conclusions

Our present study investigated the dyeing and fastness properties of *N*-methylphthalimide-based azo disperse dyes on cellulose diacetate. *N*-methylphthalimidylazo disperse dyes showed superior wash fastness properties as compared to conventional 4-aminoazobenzene disperse dyes. These results are probably attributable to the excellent wash-off properties resulting from the easy clearability imparted by the ring opening and ionization of phthalimide ring structure. Excellent wash fastness was exhibited in soaping and non-treated and hot rinsed aftertreatment samples, presumably because the phthalimide groups of the synthesized dyes were hydrolyzed to solubilizing diester groups during aftertreatment. The dye residues were, therefore, more readily removed during the wash-off and exhibited low staining on adjacent multifiber fabric in the wash fastness test. These findings are consistent with the hydrolysis analysis results using HPLC and a UV-visible spectrophotometer in the previous studies [10].

*N*-methylphthalimide-based azo disperse dyes showed relatively lower build-up properties probably because of their low tinctorial strength and affinity to acetate fibers. With regard to hydrophobicity/hydrophilicity balance, the dyes with low hydrophobicity exhibited better build-up properties on cellulose diacetate fibers. This is presumably as a result of the relatively less hydrophobic nature of cellulose acetate fibers as compared to that of PET.

Results of our present study demonstrate *N*-methylphthalimidylazo disperse dyes as a promising, efficient alternative to high value-added dyes that can be used for high color fastness dyeing of cellulose diacetate with a minimal discharge of wastewater if their build-up properties are improved to a satisfactory level.

## Figures and Tables

**Table 1:** The synthesized dyes used in the present study.

Dye Structures	Dye	R^1^	R^2^	R^3^
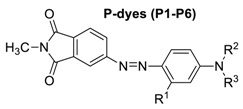 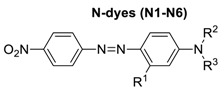	P1/N1	H	C_2_H_5_	C_2_H_5_
	P2/N2	H	C_2_H_5_	C_2_H_4_OH
	P3/N3	H	C_2_H_4_OH	C_2_H_4_OH
	P4/N4	CH_3_	C_2_H_5_	C_2_H_5_
	P5/N5	CH_3_	C_2_H_5_	C_2_H_4_OH
	P6/N6	CH_3_	C_2_H_4_OH	C_2_H_4_OH

**Table 2:** The spectral properties of the synthesized dyes.

Dye	$\lambda_{max}^{EtOH}$ (nm)	$\varepsilon_{max}^{EtOH}$ (Lmo^−1^cm^−1^)	$∆\lambda_{1/2}^{EtOH}$ (nm)	Dye	$\lambda_{max}^{EtOH}$ (nm)	$\varepsilon_{max}^{EtOH}$ (Lmo^−1^cm^−1^)	$∆\lambda_{1/2}^{EtOH}$ (nm)
P1	485	21,400	115	N1	486	35,800	125
P2	482	32,700	113	N2	485	37,300	129
P3	477	34,400	109	N3	486	35,800	134
P4	498	17,000	109	N4	499	33,300	131
P5	486	29,800	118	N5	486	33,500	138
P6	488	33,400	106	N6	488	35,800	125

**Table 3:** Colorimetric data and visual appearance of dyed cellulose diacetate fabrics with P-dyes and N-dyes at 1.0 %omf dye concentration.

Dye	L*	a*	b*	C*	*h_ab_*	Color on Cellulose Acetate
P1	62.9	47.3	42.9	63.8	42.2	Strong reddish orange
P2	54.6	55.0	53.9	77.0	44.4	Vivid reddish orange
P3	52.2	57.0	56.1	80.0	44.5	Vivid reddish orange
P4	68.2	37.5	18.3	41.7	26.0	Strong yellowish pink
P5	58.4	48.4	29.1	56.5	31.0	Deep yellowish pink
P6	59.1	49.6	40.4	64.0	39.1	Strong reddish orange
N1	48.9	55.0	39.5	67.7	34.0	Vivid reddish orange
N2	42.4	57.0	39.7	69.5	34.8	Vivid red
N3	45.0	57.5	42.9	71.7	36.8	Vivid red
N4	43.2	53.0	24.5	58.4	24.8	Strong red
N5	33.5	49.8	25.5	55.9	27.1	Vivid red
N6	35.8	51.3	28.3	58.6	28.9	Vivid red

**Table 4:** Color photographs of cellulose diacetate fabrics dyed with the synthesized dyes.

Dyes	Dye Concentration (%omf)										
	0.5	1.0	2.0	4.0	6.0		0.5	1.0	2.0	4.0	6.0
P1	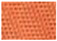	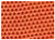	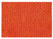	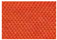	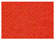	N1	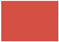	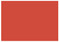	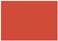	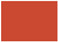	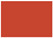
P2	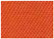	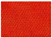	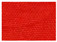	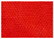	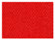	N2	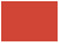	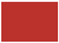	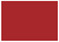	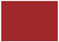	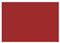
P3	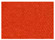	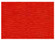	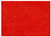	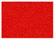	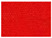	N3	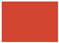	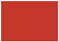	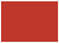	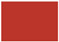	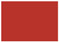
P4	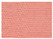	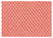	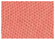	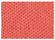	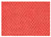	N4	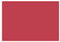	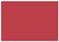	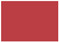	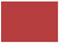	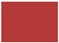
P5	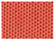	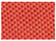	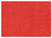	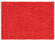	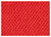	N5	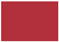	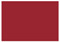	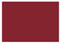	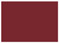	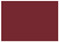
P6	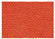	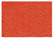	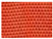	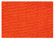	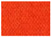	N6	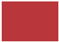	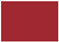	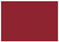	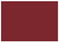	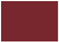

**Table 5:** Fastness to washing of dyed cellulose diacetate fabrics with dyes P3 and N3 (ISO 105 C06 D3S) at 1/1 standard depth dyeing.

Dye	After-Treatment	Staining						Change
		Wool	Acryl	PET	Nylon	Cotton	Acetate	
P3	No-treatment	4-5	5	4-5	4-5	4-5	4	4-5
	Hot-rinse	5	5	4-5	4-5	4-5	4	4-5
	Soaping	5	5	5	4-5	5	4-5	4-5
N3	No-treatment	4	4-5	4-5	2-3	4	2-3	2-3
	Hot-rinse	4	4-5	4-5	2-3	4	2-3	2-3
	Soaping	4-5	4-5	4-5	2-3	4-5	2-3	2-3

## Abbreviations

(IR) w = weak; m = medium; s = strong (^1^H NMR) s = singlet; d = doublet; q = quartet; m = multiplet.
